# The Role of Neurotrophin Signaling in Age-Related Cognitive Decline and Cognitive Diseases

**DOI:** 10.3390/ijms23147726

**Published:** 2022-07-13

**Authors:** Tadahiro Numakawa, Haruki Odaka

**Affiliations:** 1 Department of Cell Modulation, Institute of Molecular Embryology and Genetics, Kumamoto University, Kumamoto 860-0811, Japan; numakawa.yyrmk@gmail.com; 2 Cellular and Molecular Biotechnology Research Institute, National Institute of Advanced Industrial Science and Technology (AIST), Central 6, Tsukuba 305-8566, Japan

**Keywords:** brain-derived neurotrophic factor, aging, Alzheimer’s disease, intracellular signaling, exercise

## Abstract

Neurotrophins are a family of secreted proteins expressed in the peripheral nervous system and the central nervous system that support neuronal survival, synaptic plasticity, and neurogenesis. Brain-derived neurotrophic factor (BDNF) and its high affinity receptor TrkB are highly expressed in the cortical and hippocampal areas and play an essential role in learning and memory. The decline of cognitive function with aging is a major risk factor for cognitive diseases such as Alzheimer’s disease. Therefore, an alteration of BDNF/TrkB signaling with aging and/or pathological conditions has been indicated as a potential mechanism of cognitive decline. In this review, we summarize the cellular function of neurotrophin signaling and review the current evidence indicating a pathological role of neurotrophin signaling, especially of BDNF/TrkB signaling, in the cognitive decline in aging and age-related cognitive diseases. We also review the therapeutic approach for cognitive decline by the upregulation of the endogenous BDNF/TrkB-system.

## 1. Introduction

Neurotrophins consist of nerve growth factor (NGF), brain-derived neurotrophic factor (BDNF), neurotrophin-3 (NT-3), and neurotrophin-4 (NT-4) [1,2,3,4]. Each neurotrophin has a specific receptor: NGF binds to TrkA, BDNF and NT-4 bind to TrkB, and NT-3 binds to TrkC [1]. Neurotrophins are distributed among the peripheral nervous system and the central nervous system (CNS), and their critical roles in the neuronal plasticity and maintenance of cell survival have been well studied. Importantly, BDNF and its high affinity receptor TrkB are extensively and intensively expressed in a variety of brain regions, including the cortical and hippocampal areas, which are essential for learning and memory. On the other hand, the cholinergic neurons in the basal forebrain are recognized as an NGF-sensitive cell population in which TrkA (NGF specific receptor) and p75NTR (common for all neurotrophins) are expressed.

The activation (phosphorylation) of TrkB, induced by binding with BDNF, stimulates downstream intracellular signaling pathways, mainly, resulting in the positive regulation of cell differentiation, survival, maturation, and synaptic function [5,6]. Importantly, the BDNF/TrkB system contributes to neurogenesis in the embryonic and adult stages [7]. These TrkB-mediated signaling transductions and resultant cellular events are caused by mature (processed) BDNF. Evidence demonstrates that the BDNF molecule is first translated as a precursor protein (proBDNF), and subsequently cleaved to the small mature protein, which has a high affinity for the TrkB receptor. On the other hand, the precursor proneurotrophins (including proBDNF) bind to p75NTR with high affinity, while all mature neurotrophins are associated with p75NTR with low affinity. Studies showed that the p75NTR-mediated signaling has negative roles in neuronal aspects, including cell survival and synaptic plasticity [8,9,10].

As expected, studies have demonstrated the contribution of an alteration in neurogenesis and deficits in synaptic function to the pathophysiology of neurodegenerative diseases and aging; therefore, it is required to understand the detail of neurotrophin action in the CNS, in order to establish an effective therapeutic candidate. In this review, we introduce current evidence regarding the relationship among neurotrophins (especially, BDNF, NGF, and their receptors) and the disrupted neuronal function and related brain diseases.

## 2. Intracellular Signaling by Neurotrophins

**Figure 1:**
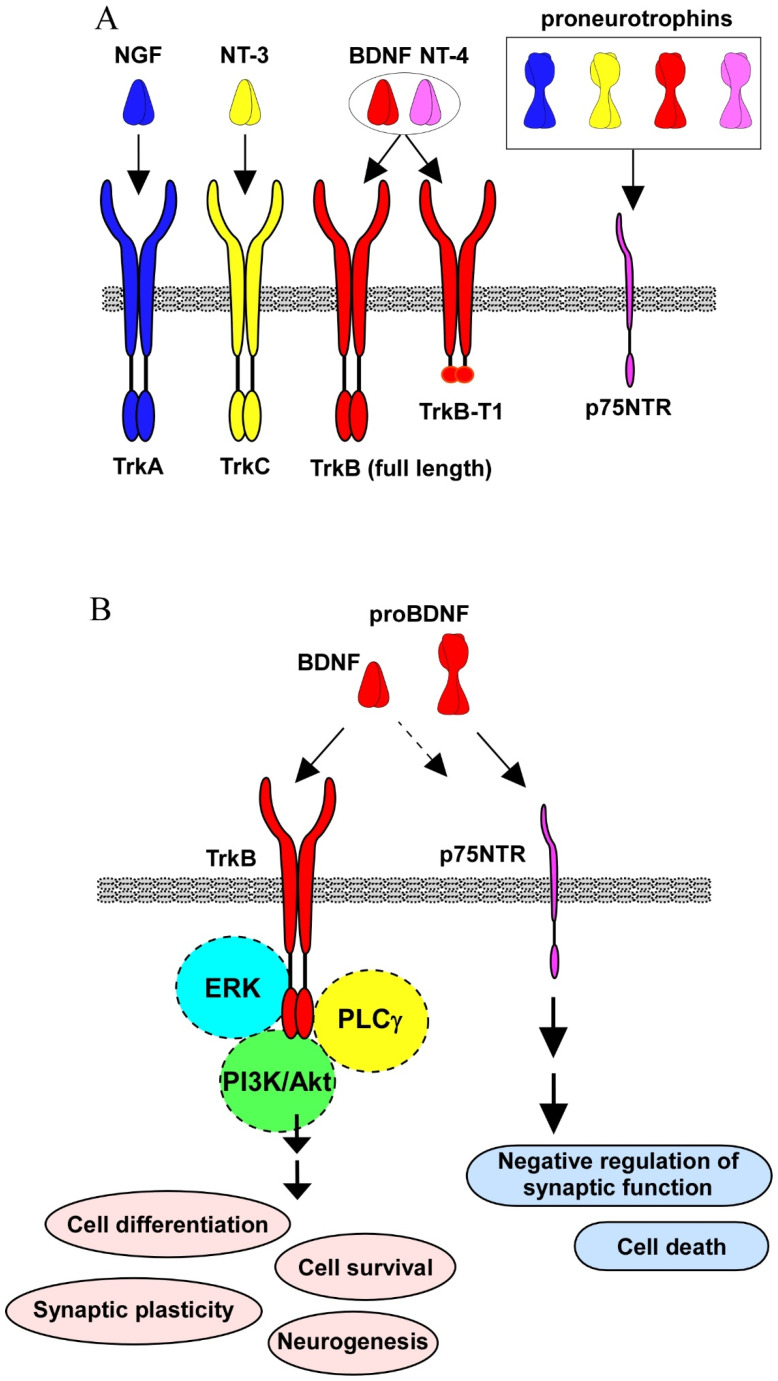
Neurotrophins, receptors, and intracellular signaling. (**A**) Neurotrophins consist of nerve growth factor (NGF), brain-derived neurotrophic factor (BDNF), neurotrophin-3 (NT-3), and neurotrophin-4 (NT-4). Each neurotrophin has high affinity receptor; NGF binds to TrkA, BDNF and NT-4 to TrkB, and NT-3 to TrkC. In addition, all neurotrophins bind to p75NTR with low affinity. Mature (processed) neurotrophin is firstly translated as a precursor proneurotrophin which has high affinity for p75NTR. (**B**) Activation of TrkB stimulated by BDNF triggers downstream intracellular signaling pathways (mainly, PI3K/Akt, ERK, and phospholipase Cγ (PLCγ) contributes to positive regulation of cell differentiation, survival, synaptic function, and neurogenesis. p75NTR-mediated signaling is involved in negative regulation of cell survival and synaptic function. Truncated isoform TrkB.T1 exerts a dominant-negative role against the function of full-length TrkB or a BDNF scavenging effect in the CNS.

Three signaling pathways, including PI3k/Akt, ERK, and phospholipase Cγ (PLCγ) are activated after BDNF binding to TrkB [1]. In the CNS neurons, it was confirmed that the promotion of cell differentiation and survival, enhancement of synaptic function, maintenance of neurogenesis, and neuroprotection against cell death under the sever condition, such as oxidative stress, are dependent on the activation of these intracellular signalings. For example, it is well demonstrated that Akt-signaling contributes to neuronal survival, and an intracellular Ca^2+^ increase through the PLCγ pathway is important for synaptic function, including neurotransmitter release in glutamatergic neurons (see Figure 1) [1]. Interestingly, ERK-signaling exerts a positive and/or negative impact on the CNS (see Figure 1). In general, the Akt signaling pathway has been mainly found to promote the cell survival of a number of neuronal cell types; however, ERK signaling is required for a variety of neuronal aspects. We previously reported ERK-dependent upregulation of synaptic proteins that are essential for neurotransmitter release [11]. In addition, a growing number of studies have shown neuronal cell death by the activation of ERK signaling (see Rai et al., 2019 [12]).

It has been accepted that TrkB truncated isoform receptors can be transducers, determining the action of BDNF. Evidence shows that the TrkB gene encodes a number of isoforms, including TrkB.T1, one of isoforms lacking the catalytic tyrosine kinase domain [13]. In addition to TrkB full-length (for Akt-, PLCγ-, and ERK-signaling pathways), the truncated isoform TrkB.T1 elicits intracellular signaling independently, and is involved in the pathogenesis of neurological disorders, including AD, Parkinson’s disease, stroke, mood disorders, and schizophrenia (see the review by Tessarollo and Yanpallewar, 2022 [13]). The most famous mechanisms of TrkB.T1 contribution to BDNF action are a dominant-negative role against TrkB full-length function, or a BDNF scavenging effect. In addition to full-length receptors, truncated TrkB.T1, TrkC.T1, and TrkB.Shc were reported as the most biologically relevant splice variants [14]. TrkB.T1 was expressed in the heart, kidney, pancreas, and brain. In contrast, it has been reported that TrkB.Shc was expressed in the brain. As a function of truncated Trks, Michaelsen et al. (2010) reported that TrkB.T1 overexpression animals exhibited significant deficits in the long-term potentiation (LTP, one of the several form of synaptic plasticity) and depression [15]. Interestingly, an increase in neuromuscular performance and nerve-evoked muscle tension in TrkB.T1 null mice was reported [16]. In the TrkB.T1 null muscle, activation of full-length TrkB and Akt stimulated by the contractile activity was increased [16]. Furthermore, in the hSOD1(G93A) ALS mice, the deletion of TrkB.T1 induced the delayed onset of motor neuron degeneration, without a change of the life span of the animals [17].

In addition to the interaction between full-length- and truncated-Trks, p75NTR also affects Trks signaling. Intracellular signaling through p75NTR was demonstrated, although p75NTR’s cytoplasmic domain does not have catalytic activity [18]. Studies have shown p75NTR association with a variety of interactors [19,20,21]. For example, p75NTR-associated cell death executor (NADE), neurotrophin receptor interacting factor 1 and 2 (NRIF1 and 2), and neurotrophin receptor-interacting melanoma antigen homolog (NRAGE) have been reported (see the review by Becker et al., 2018 [21]). Tumor necrosis factor receptor-associated factors (TRAF) family proteins also interact with p75NTR, regulating the nuclear factor-kB pathway [22]. Yamashita et al. (1999) found an interaction of p75NTR with the Ras homolog gene family member A and observed an increase in the neurite elongation via the interaction [23]. Importantly, sortilin acts as a co-receptor for proNGF, and an interaction of p75NTR and sortilin is essential for cell death caused by proNGF [24]. Further, sortilin recognizes the pro-domain structure of proBDNF and functions as a partner for p75NTR in cell death induction [25,26]. Furthermore, the JNK pathway has also been demonstrated as a contributor in cell death caused by proNGF [27]. On the other hand, NF-κB signaling, stimulated by the proNGF-p75NTR interaction, has a role in cell survival [28]. Considering the diversity of receptor types (TrkB full-length, truncated isoform, and p75NTR), neurotrophin forms (precursor or mature form), and their diverse distribution in the brain regions, further cellular/molecular information regarding neurotrophin biology in the target region is required to stimulate specific signaling for the treatment of brain diseases.

## 3. Neurotrophin, Synaptic Function, and Neurogenesis

BDNF is an extensively examined neurotrophic factor regarding synaptogenesis and synaptic plasticity. BDNF affects both pre- and post-synaptic machinery via a variety of mechanisms [29,30]. Therefore, it is well-established that BDNF is a critical factor for the formation and maintenance of memory function of the brain via regulating synaptic consolidation [31,32]. Such an importance of BDNF in synaptic regulation prompts scientist to approach its potential as a candidate to treat psychiatric disorders in which the dysregulation of synaptic function occurs [33]. Indeed, a growing amount of evidence has suggested that downregulation of BDNF levels and/or function is related to the pathophysiology of psychiatric disorders (see Numakawa et al., 2013 [34]).

Concerning the dendritic growth in the developing visual cortex, neuronal responses to all neurotrophins, including NGF, BDNF, NT-3, and NT-4, were examined [35]. Interestingly, when examining an increase in the length and complexity of dendrites, basal dendrites of pyramidal neurons in layer 4 responded to BDNF; in contrast, neurons in layers 5 and 6 responded to NT-4, suggesting that the actions of the TrkB ligands, BDNF, or NT-4 were distinct [35]. Distinct signaling/functions are demonstrated in the neurons by acute and gradual increases in the concentration of BDNF [36]. An acute increase in BDNF caused the transient activation of TrkB in cultured hippocampal neurons, although a sustained activation of TrkB was induced by a gradual increase in BDNF concentration. Interestingly, BDNF promoted neurite elongation and spine head enlargement after transient TrkB activation. In contrast, sustained TrkB activation induced neurite branching and spine neck elongation [36]. TrkB activation potentiates the evoked frequency and amplitude in both slice and dissociated neuronal cells. In hippocampal slices, influences of NGF, BDNF, or NT-3 on the synaptic transmission were investigated [37]. Exogenous BDNF or NT-3 induced a dramatic and sustained potentiation of synaptic strength at the Schaffer collateral-CA1 synapses; however, NGF failed to do so. The positive action by BDNF or NT-3 was abolished by K252a (an inhibitor for kinase activity of Trks) application. Reduced paired-pulse facilitation (one of the presynaptic events) was induced by both NT-3 and BDNF [37]. An increased frequency and amplitude of excitatory postsynaptic currents after BDNF application in dissociated hippocampal neurons was also reported [38]. In general, it is considered that an influence on the frequency of postsynaptic currents is resulted from the changed presynaptic function, and the effect on amplitude is a postsynaptic one. Importantly, the increased amplitude was selectively inhibited by K-252a, indicating TrkB-mediated postsynaptic regulation [38]. Recently, to clarify the mechanism of the BDNF-mediated LTP, a deletion of BDNF and TrkB in the CA1 and CA3 hippocampal region has been performed using a viral-mediated approach [30]. The deletion of BDNF and its receptor at pre- and/or postsynaptic sites revealed that presynaptic BDNF was involved in LTP induction, and postsynaptic BDNF was required for LTP maintenance. Furthermore, it was also revealed that presynaptic TrkB had a role in maintaining LTP, although the LTP induction required postsynaptic TrkB [30].

Remarkedly, neurogenesis is also affected by BDNF. Both embryonic and adult neurogenesis have critical roles in the normal establishment and function of the nervous system [39]. Embryonic neurogenesis has several steps: the proliferation/differentiation of neural progenitor cells (NPCs), migration of neurons to the proper position, and synaptogenesis. Using BDNF- or TrkB knockout mice, it has been discovered that the BDNF/TrkB system is essential for embryonic neurogenesis and an establishment of a normal nervous system (summarized in Numakawa and Odaka, 2021 [7]). The essential role of neurotrophins in neurogenesis has been investigated in a variety of animal models, including primates, rodents, and zebrafish. Although mammalian models are the main focus of this review, an interesting link between abundant neurotrophic signaling in neurogenic niches and the regenerative capacity of the zebrafish brain has been summarized by Cacialli [40]. Importantly, the neurogenesis following the development stage, which persists throughout life in the hippocampal dentate gyrus (DG) and the subventricular zone (SVZ), is called adult neurogenesis, and the neurogenesis is also regulated by BDNF. Recent studies have suggested the possible involvement of the alteration in the neurogenesis of the pathophysiology of neurodegenerative diseases and aging [41].

BDNF influences granule cells in the hippocampal dentate gyrus by affecting neurogenesis. Scharfman et al. (2005) investigated changed hippocampal neurogenesis in rats with or without BDNF, using osmotic pumps (2-week infusion into the hippocampus) implanted unilaterally in the dorsal hilus. One month after the infusion ended, they performed immunocytochemical analysis using antibodies to BrdU (administered twice daily during the 2-week infusion period), and a neuronal nuclear protein (NeuN). The number of BrdU(+)/NeuN(+) double-positive granule cells were increased by BDNF infusion [42]. Further, using knockdown of BDNF with RNA interference and lentiviral vectors injection, Taliaz et al. (2010) found reduced BDNF expression in the DG and significant decreased neurogenesis [43]. It was demonstrated that chronic application of antidepressants or wheel-running exercise failed to activate neurogenesis when the ablation of TrkB was conducted [44]. Remarkably, they also found that the mice lacking TrkB only in the differentiated DG neurons respond normally to chronic antidepressants and displayed an increased neurogenesis [44], suggesting a critical role of TrkB in regulating hippocampal neurogenesis. Recently, it has been recognized that antidepressants, including selective serotonin reuptake inhibitors and physical exercise, promote adult neurogenesis and are able to counteract depressive behaviors [45]. It is possible that an upregulation of BDNF and/or TrkB is a beneficial target to improve the behavior of brain disorders displaying impaired neurogenesis.

Previously, we reported that decreased GR expression caused by chronic glucocorticoid exposure, and the downregulation of GR, is involved in the suppression of BDNF-mediated neurotransmitter release [46]. Indeed, studies have suggested that a crosstalk between BDNF/TrkB and GR systems is involved in stress-related disorders, including depression [47]. Because of its easy control, analgesia, a muscle relaxer, and sevoflurane, an anesthetic, are commonly used for elderly patients. However, sevoflurane has a risk of causing perioperative neurocognitive disorders (PND). Xu et al. (2022) showed that a 3% sevoflurane exposure induced cognitive impairment and the inhibition of adult hippocampal neurogenesis in the DG of aged (18-month-old) mice, but not adult (8-month-old) mice [48]. Consistently, downregulation of BDNF/TrkB and NT-3/TrkC were observed due to treatment with 3% sevoflurane. As expected, hippocampal BDNF or NT-3 microinjection partially improved the cognitive impairment and decreased hippocampal neurogenesis caused by sevoflurane, suggesting that a downregulation of these neurotrophins contributes to the onset of PND [48].

Of course, the classical approach to protect mature neurons against cell toxicity is important, and application of natural products has been also conducted. Therefore, to achieve an upregulation of BDNF to improve behavior of both psychiatric and neurodegenerative disorders, targeting the BDNF/TrkB system with natural products has been prioritized [49]. A mice model of depression established by chronic treatment with corticosterone, a stress hormone, showed decreased hippocampal neurogenesis [50]. Interestingly, the application of formononetin (FMN), a type of isoflavone, improved the reduced neurogenesis caused by chronic corticosterone treatment [50]. Moreover, upregulation of the glucocorticoid receptor (GR) and BDNF was observed after the FMN treatment, in addition to an improvement in sucrose preference and a decrease in the immobility time in the forced swimming test in the animal model, suggesting that BDNF action stimulated by FMN is effective against depressive behaviors.

It has been reported that Samhwangsasim-tang (SST), an herbal medicine complex containing Coptidis Rhizoma, Scutellariae Radix, and Rhei Rhizoma, exerts neuroprotective effects [51]. In the hippocampal cell death model, SST treatment reduced cell death. Moreover, cognitive impairment of mice establishment by intraperitoneally injected scopolamine was improved by SST pre-administration. Importantly, BDNF, TrkB, and cAMP-response element binding protein (CREB) activation were all upregulated, while downregulation of the p75NTR was observed after SST treatment, suggesting that stimulation of the BDNF/TrkB system is involved in the neuroprotection effect of the herbal medicine complex [51]. It has been also shown that an activator of the voltage-gated sodium channel, antillatoxin, a lipopeptide isolated from the cyanobacterium Moorea producens, promoted the neurite outgrowth of murine cerebrocortical neurons [52]. Interestingly, the release of BDNF, and the resultant BDNF-stimulated TrkB/Akt signaling, contributed to the antillatoxin-induced neurite outgrowth because inhibitors of TrkB or Akt signaling prevented the effect of antillatoxin.

In comparison with the action of BDNF-related agonists against neural toxicity, the potential effectiveness of TrkA (or NGF) agonists has not been adequately demonstrated. Rogdakis et al. has shown that ENT-A013, a novel NGF mimetic, selectively activates the TrkA receptor [53]. Due to NGF’s low bioavailability and its impermeability through the blood–brain-barrier, it has been considered that the use of NGF as a potential therapeutic agent against neurodegenerative diseases, including AD, was limited. ENT-A013, a synthetic dehydroepiandrosterone derivative, was selected to examine its neuroprotective effect through TrkA stimulation, and it was found that the NGF mimetic exerted a protective effect against amyloid β-induced apoptosis in cultured hippocampal neurons. In addition, decreased long-term potentiation (a typical synaptic plasticity) caused by amyloid β application was significantly restored by the treatment with ENT-A013. As shown above, a variety of activators for the BDNF/TrkB system were well examined, and the corroboration with such an agonist for TrkA is very interesting to approach the therapeutic strategy against neurodegenerative diseases. A recent study has demonstrated that action balance of proNGF/NGF is critical for poststroke neurological rehabilitation. Using cerebral ischemia-reperfusion (CIR) models with PC12 cell lines or rats, Li et al. found that proNGF was predominant within 24 h reperfusion, followed by mature NGF production from the proNGF [54]. As expected, the mature NGF had a neuroprotective effect against autophagic and apoptotic damage after the CIR; however, proNGF contributed to both autophagic and apoptotic processes. In their system, the PI3K/Akt/mTOR and ERK pathway contributed to the neuroprotection through a mature form of NGF, while the proNGF stimulated the ERK pathway, resulting in increasing autophagy and apoptosis [54]. Thus, a TrkA-specific agonist (for example, ENT-A013) may be beneficial for neuroprotection against proNGF/p75NTR-dependent cell damage after CIR.

## 4. Aging, Neurotrophin Signaling, and Neuroinflammation

Cognitive function gradually decreases with normal aging. It has been reported that the frontal cortex and the hippocampus were the brain regions most vulnerable to aging, as evidenced by the continuous reduction in volume and functional dysfunction in memory and cognition with normal aging [55]. Age-related volume reduction is accompanied with altered dendritic branching patterns, reduced dendritic spines, and a decrease in adult neurogenesis. Because of the important roles of BDNF in synaptic plasticity, neurogenesis, and learning and memory function, a number of studies investigated the involvement of BDNF-mediated signaling in age-related cognitive decline. It has been reported that TrkB mRNA expression in the human dorsolateral prefrontal cortex is reduced in the elderly, in comparison with young adults [56]. Hippocampal BDNF mRNA expression was not significantly changed with age, whereas TrkB and TrkB.T1 mRNA levels were decreased over the life span [57]. Oh et al. examined microarray data from the orbitofrontal cortex of 209 healthy subjects, ranging from 16–96 years old, and confirmed the age-related downregulation of BDNF gene expression [58]. Both excitatory and inhibitory synaptic genes were also downregulated with age and positively correlated with BDNF and TrkB expression, while negatively correlated with TrkB.T1 level. In addition, a three weeks blockage of TrkB activity in adult mice induced an aging-related transcriptional pattern in the expression of markers for inhibitory presynaptic genes in the frontal cortex [58]. The administration of BDNF also directly affects the cognitive function in aged animals. Aged rats treated with 28-day infusions of BDNF protein into the medial entorhinal cortex showed an improvement in spatial memory [59]. In addition, the lentiviral gene delivery of *BDNF* into the entorhinal cortex restores age-related impairment of visuospatial learning in aged rhesus monkeys [59]. ProBDNF may also contribute to age-related memory impairment. ProBDNF levels were increased in the hippocampus of aged mice in comparison with young mice [60]. In addition, infusions of proBDNF into the CA1 region of the dorsal hippocampus cause a progressive impairment of memory performance, which is accompanied by an increased level of p-cofilin, an important regulator of dendritic spines [60]. In contrast, intra-hippocampal infusions of TAT-Pep5, which blocks the interaction between p75NTR and RhoGDI, improved learning and memory function [60]. Considering the conflicting effect of proBDNF/p75NTR and BDNF/TrkB signaling, the imbalance of two signaling cascades may contribute to age-related cognitive decline. Collectively, these findings indicate the protective role of BDNF against cognitive decline with normal aging.

Several studies also reported the alternation of NGF signaling with aging [61]. NGF is produced in the cortical/hippocampal regions, and it supports the survival and neural plasticity of cholinergic neurons in the basal forebrain [62]. Lärkfors et al. showed that aged rats exhibited a 40% decrease in NGF protein levels in the hippocampus and a 50% decrease in NGF mRNA levels in the forebrain regions (cerebral cortex, hippocampus, basal forebrain, and hypothalamus) in comparison with young adult Fischer 344 rats [63]. Although aged Wistar rats did not show a reduction in NGF mRNA levels in the hippocampus, TrkA mRNA expression in the basal forebrain and the caudate were found to be decreased [64]. It has also been reported that aged Long Evans rats had decreased levels of NGF and phospho-TrkA receptors, but an increased expression of proNGF, p75NTR and sortilin in the prefrontal cortex and hippocampus, as well as deficits in recognition memory and spatial memory [65]. Al-Shawi et al. also showed that proNGF levels were increased in the hippocampus of aged mice and rats [66]. Treatment with proNGF induces neural death in the primary culture of the basal forebrain and peripheral sympathetic neurons of old, but not of young, adult rodents [66]. The inhibition of p75NTR and sortilin interaction by neurotensin treatment prevented proNGF-induced cell death in cultured neurons from aged rodents, suggesting a critical role of sortilin in age-related proNGF neurotoxicity [66]. Because expression of sortilin is increased in the basal forebrain and sympathetic neurons of aging rodents, upregulation of the proNGF/p75NTR/sortilin system may contribute to age-related neurodegeneration in the cholinergic populations [66]. Enhancement of proNGF signaling may also impair the adult neurogenesis in the hippocampus. Guo et al. reported that proNGF induced cell cycle arrest in the G0/G1 phase and inhibited proliferation of NSCs isolated from a postnatal mouse hippocampus [67]. The inhibitory effect on neurogenesis was reversed by the fusion protein of p75NTR extracellular domain and human IgG Fc fragment (p75NTR/Fc), and by knockout of p75NTR [67]. In addition, proNGF treatment inhibits ERK 1/2 phosphorylation, and an inhibitor for ERK1/2 mimics the effect of proNGF treatment, suggesting a contribution of ERK1/2 inhibition via proNGF/p75NTR signaling on neurogenesis [67]. These studies suggest the contribution of NGF/proNGF signaling disturbance to age-related cognitive decline.

Growing evidence indicates the critical interaction between neuroinflammation and neurotrophin signaling in age-related cognitive defects. Aging shifts the state of microglia, brain-resident macrophage, from a quiescent state to primed one. Primed microglia in an aged brain release more proinflammatory cytokines, including IL-1β, IL-6, and TNFα in a prolonged manner upon immune challenge, which leads to chronic neuroinflammation [68]. There are several animal models of peripheral immune challenge induced microglial activation accompanied with downregulation of BDNF and/or TrkB expression in the aged brain. Cortese et al. found that peripheral *E. coli* infection induced memory impairment and decreased BDNF protein in hippocampal synaptoneurosomes in aged, but not young, rats [69]. The phosphorylation level of TrkB and its downstream signal molecules ERK and PLC-γ were also attenuated in aged hippocampal synaptoneurosomes [69]. In addition, the suppression of BDNF/TrkB signaling by the infection was reversed by administration of the anti-inflammatory cytokine IL-1Ra (interleukin 1-specific receptor antagonist), suggesting a causative role of IL-1β in the infection-evoked reduction in BDNF at the hippocampal synapses [69]. Postoperative cognitive dysfunction (POCD) is a cognitive impairment in patients following anesthesia and surgery and is more common in the elderly. Anesthesia and surgery in aged rats induce microglia activation, increased proinflammatory cytokines, and downregulation of BDNF and synaptic protein in the hippocampus [70]. Cisterna magna infusion of anti-inflammatory cytokine IL-4 attenuated these abnormalities and improved the spatial memory in POCD model rats [70]. Qiu et al. also showed that aged mice that were subjected to exploratory laparotomy with isoflurane anesthesia exhibited increased proinflammatory cytokines (IL-1β and IL-6), decreased BDNF and TrkB protein, hippocampal dendritic spine loss, and impairment in contextual fear memory [71]. They also found that TrkB was abnormally truncated into a catalytically inactive form by Ca^2+^-dependent proteases calpain in POCD mice. The over-activation of calpain was induced by Ca^2+^ influx via the Ca^2+^-permeable glutamate receptor NMDAR. Treatment with the NMDAR inhibitor or calpain inhibitor restored behavioral and cellular abnormalities in POCD model mice, suggesting a contribution of the NMDAR/Calpain/TrkB cascade on POCD pathology [71]. A recent study also highlights the key role of the BDNF/proBDNF balance on the POCD pathology [72]. POCD model mice showed an increase in proBDNF and phosphorylated p75NTR levels and a decrease in BDNF and phosphorylated TrkB levels, resulting in a marked decrease in the BDNF/proBDNF ratio in the hippocampus. Exogenous BDNF or p75NTR inhibitor treatment recovered the reduction in the dendritic spine and the impairment in synaptic plasticity and fear conditioning memory [72].

## 5. Proinflammatory Cytokine and BDNF Signaling

Consistent with the BDNF/TrkB downregulation in aged animals treated with immune challenge, it has been also demonstrated that proinflammatory cytokine IL-1β affects the BDNF/TrkB signaling cascade. The hippocampal infusion of IL-1β in rat blocks contextual fear conditioning-induced BDNF upregulation, which is essential for memory consolidation [73]. It has also been reported that subacute (8 days) intracerebroventricular administration of IL-1β induced memory defects and a reduction in BDNF and TrkB protein in the rat hippocampus [74]. In addition to a reduction in BDNF and TrkB expression, IL-1β also affects the downstream cascade of BDNF/TrkB signaling. In rat organotypic hippocampal cultures, IL-1β treatment suppressed the BDNF-induced upregulation of Arc and phosphorylation of cofilin and CREB, which were essential event for the stabilization of synaptic plasticity [75]. IL-1β impaired the phosphorylation of insulin receptor substrate 1, a protein that mediates the PI3K/Akt signaling cascade, and which is also known to be activated when BDNF stimulates TrkB. These inhibitory effects on BDNF/TrkB signaling were reversed by the p38 MAPK inhibitor, suggesting a pivotal role of p38 MAPK signaling in these IL-1β-induced events [75]. These studies suggested that enhanced neuroinflammation in aged brains leads to the downregulation of BDNF/TrkB signaling via increasing proinflammatory cytokine.

## 6. BDNF and NGF Signaling in Alzheimer’s Disease

Aging is a major risk factor of cognitive disease, and dysregulation of BDNF/TrkB signaling has been also reported in age-related cognitive disorders. Alzheimer’s disease (AD) is the most common dementia, and it is characterized by the formation of neuritic plaques composed of amyloid β and neurofibrillary tangles consisting of hyperphosphorylated tau protein. Accumulated amyloid β and/or hyperphosphorylated tau results in an impairment of synaptic plasticity, neuroinflammation, and neuronal cell loss. Increasing evidence indicates a critical role of BDNF in AD pathology. BDNF and proBDNF protein levels were decreased in the human parietal cortex of subjects with mild cognitive impairment (MCI) and AD patients [76]. The BDNF level in cerebrospinal fluid was also reduced in AD patients compared to MCI and healthy controls, and such decreased BDNF levels are significantly associated with progression from MCI to AD [77]. In a mice model of AD, which has the human amyloid precursor protein (APP) transgene bearing both the Swedish and the Indiana APP mutations, BDNF gene delivery in entorhinal cortices by lentiviral vectors restored synapse loss, ERK phosphorylation, hippocampus-dependent memory, and learning function [59]. Although lentiviral BDNF gene delivery to mice after disease onset failed to mitigate neuronal cell loss, lentivirus administration before disease onset rescued it [78]. In both cases, *BDNF* gene delivery before and after disease onset, the amyloid β plaque in AD model mice was not affected, suggesting that the beneficial effects of BDNF are independent from the toxicity of insoluble amyloid β [59,78]. AD mouse models carrying human APP transgene with Swedish double mutation (KM670/671NL) and human PS1 transgene with L166P mutation (APP/PS1-mice) were crossed with heterozygous *BDNF* knockout (BDNF+/−) mice [79]. APP/PS1-BDNF+/−-mice exhibited an accelerated learning impairment in a two-way active avoidance task, without any change of amyloid β plaque levels, in comparison to APP/PS1- and BDNF+/−-mice [79]. The MAPT P301L mutation was originally found in familial frontotemporal dementia patients and is used to generate a mouse model of tauopathy characterized by the aggregation of hyperphosphorylated tau protein, which is a common pathology of AD and frontotemporal dementia. The P301L transgenic mice showed decreased BDNF expression in the brain [80]. Intralateral ventricle injection of adeno-associated virus carrying the human *BDNF* gene restored neuronal degeneration, synaptic loss, and cognitive decline in the Morris water maze, Y-maze, and novel objects recognition tests but did not reverse the tau hyperphosphorylation level [80]. Therefore, the BDNF downregulation likely occurred downstream of amyloid β and tau pathology, showing direct linkage with the decline in memory and learning functions.

Because of the severe neurodegeneration of the cholinergic neurons in the basal forebrain and linkage with cholinergic input and cognitive function in AD, NGF signaling has been indicated as a key modulator of AD pathology [81]. Meta-analysis of 98 articles investigating neurotrophic factor levels in CSF and the blood of AD patients showed increased NGF levels in CSF of AD patients [82]. A systematic review covering 23 post-mortem studies of AD patients showed that most studies suggested increased proNGF levels in the hippocampus and neocortex of patients with AD [82]. As a mechanism of proNGF accumulation in the AD brain, it has been proposed that there are disturbances in the conversion of proNGF to NGF [81]. Bruno et al. (2006) demonstrated that NGF is produced in the synaptic cleft via a coordinated release of proNGF, zymogens, convertases, and endogenous regulators in an activity-dependent manner [83]. Released zymogen plasminogen was converted into its active form plasmin by the tissue plasminogen activator (tPA), and then plasmin cleaves the prodomain of proNGF. Pharmacological inhibition of plasmin leads to a reduction in NGF levels, accumulation of proNGF, cholinergic neurodegeneration, and cognitive impairment in rats [84]. An increase in plasminogen protein and neuroserpin (endogenous tPA inhibitor), and a reduction in tPA protein levels in the brain tissue of AD patients suggested the downregulation of the proNGF convertase, plasmin [85]. They also found an overactivation of matrix metalloproteinase 9, which degrades NGF protein in the synaptic cleft [85]. Therefore, suppression of proNGF conversion and enhancement of NGF degradation induces proNGF accumulation and reduced the availability of NGF, which may explain the vulnerability of the cholinergic neuron in AD patients.

## 7. Cognitive Improvement by Exercise with BDNF Signaling Upregulation

In addition to the pathological role of age-related cognitive defect, an alteration of neurotrophin signaling is also proposed as one of the mechanisms for cognitive improvement. Physical exercise has shown to improve cognitive performance in both human and animal models. Mild-intensity exercise programs improve age-related impairments in long-term spatial learning and memory, which were accompanied by the activation of Akt and CREB signaling and upregulation of BDNF mRNA and protein levels in the rat hippocampus [86]. Gomez-Pinilla et al. showed that exercise affects the epigenetic regulation of *BDNF* expression in the hippocampus [87]. Using a rat model, it has been demonstrated that DNA demethylation of neuronal activity-responsive BDNF promoter IV and activated methyl-CpG-binding protein 2 were upregulated by exercise. Exercise also increases the acetylation of histone H3 and reduces levels of the histone deacetylase 5, which was implicated in the regulation of *BDNF* gene expression [87]. The blockage of BDNF signaling in the rat hippocampus by treatment of TrkB-IgG, which scavenges endogenous BDNF, results in an inhibition of exercise-induced upregulation of CREB and synapsin I, enhancement of synaptic plasticity, and improvement in hippocampal learning and memory function, demonstrating an involvement of BDNF in the beneficial effect of exercise [88]. It has also been demonstrated that significant cognitive improvement in AD model mice was achieved by exercise. Long-term treadmill exercise for 5 months (6 sessions per week) improved spatial memory in APPswe/PS1dE9 AD mice [89]. In addition, the treadmill exercise significantly increased the number of BDNF-positive cells and decreased the proportion of activated microglia in the cerebral cortex and hippocampus of AD mice, without affecting the accumulation of β-amyloid [89]. As a molecular mechanism under the BDNF upregulation and restoration of memory function after physical exercise, a possible contribution of exercise-stimulated myokine release has been investigated. Irisin is a myokine released into the circulation by cleavage from fibronectin type III domain-containing protein 5 (FNDC5) upon physical exercise. Forced expression of FNDC5 in the primary cortical neurons increased BDNF expression [90]. In contrast, knockdown of FNDC5 caused downregulation of BDNF [90]. An in vivo study also showed that the peripheral delivery of FNDC5 with adenoviral vectors increased blood irisin and the expression of BDNF in the hippocampus of mice [90]. A recent study has demonstrated that levels of FNDC5/irisin are reduced in hippocampi and cerebrospinal fluid of AD patients and in animal AD models [91]. In addition, the blockage of irisin by intraperitoneal injections of anti-FNDC5 antibodies decreased hippocampal FNDC5/irisin levels and attenuated the protective actions of physical exercise against the impairments in synaptic plasticity and spatial memory in AD model mice. Furthermore, the peripheral delivery of FNDC5/irisin rescued memory defects in Aβ oligomer-infused mice [91]. Another blood–brain barrier permeable myokine, cathepsin B (CTSB), has also been proposed as a mediator of exercise-dependent cognitive improvement. CTSB was released from skeletal muscle cells, and elevated CTSB levels were observed in human plasma after treadmill exercise [92]. In addition, CTSB levels were correlated with fitness and hippocampus-dependent memory function [92]. Interestingly, CTSB knockout mice did not display an exercise-induced spatial memory improvement, antidepressant effect, or enhancement of adult neurogenesis in the hippocampus [92]. Moreover, recombinant CTSB treatment increased BDNF and DCX (newborn neuron marker) expression in primary hippocampal neural progenitor cells [92]. These studies demonstrate that myokine is an important mediator of beneficial effects achieved by physical exercise, and it could be a potential therapeutic target to boost BDNF expression and memory/learning function in the hippocampus.

## 8. Conclusions

**Figure 2:**
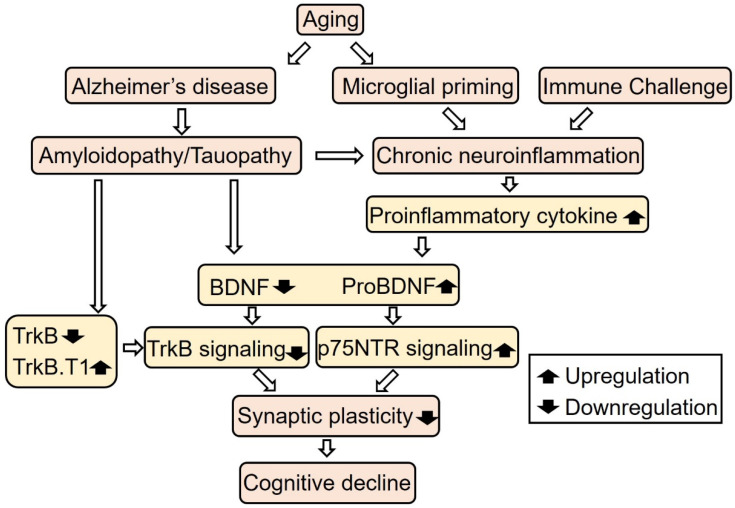
Schematic illustration of the effect of BDNF signaling disturbance on cognitive dysfunction.

In this review, we reviewed the roles of neurotrophins (especially on the BDNF/TrkB signaling) in the CNS neurons and their pathological contribution to the normal aging and age-related cognitive diseases, such as POCD and AD (Figure 2). We also introduced the beneficial effects of exercise on cognitive function and their relationship with upregulation of BDNF/TrkB signaling.

BDNF/TrkB signaling is a key regulator in synaptic plasticity in the CNS and is closely related to cognitive function. As shown by a number of studies described in this article, a weakened BDNF/TrkB signaling is a common downstream pathology in cognitive decline associated with normal aging and age-related diseases. Therefore, an enhancement of the BDNF/TrkB system would be a promising therapeutic target to treat cognitive dysfunction. However, low brain–blood barrier permeability and the short half-life of BDNF protein, as well as desensitization of TrkB by excess BDNF, limit the feasibility of BDNF administration as a therapeutic approach. On the other hand, various stimuli, such as flavonoids (see the review by Numakawa and Odaka, 2021 [93]) and exercise, are expected to potentiate endogenous BDNF/TrkB signaling. By analyzing the mechanism underlying enhanced endogenous BDNF signaling (such as myokines introduced in this review), the discovery of new therapeutic targets may be expected.

One of the major limitations of current studies on neurotrophin biology is that most of them have been investigated in rodent animal models. Because of the limitation of accessibility to human brain samples, most studies concerning cognitive decline have been limited to observational studies using relatively few postmortem brain samples. Recently, a variety of human disease models, including AD, using human iPS cell-derived neural cells and cerebral organoids have been reported [94]. These models using human neurons make it possible to perform invasive experiments, such as genetic manipulation and pharmacological experiments, that have previously only been done with animal models. In the future, it will be possible to achieve new findings in neurotrophin biology by utilizing disease models established using human cells.

Aging increases the risk of Alzheimer’s disease and neuroinflammation via microglial priming, resulting in the disbalance of BDNF/ProBDNF and/or TrkB/TrkB.T1, an impairment of synaptic plasticity, and a decline in cognitive function.

## Data Availability

Not applicable.
